# Body Composition Parameters Correlate to Depression Symptom Levels in Patients Treated with Hemodialysis and Peritoneal Dialysis

**DOI:** 10.3390/ijerph20032285

**Published:** 2023-01-27

**Authors:** Marijana Vučković, Josipa Radić, Ela Kolak, Dora Bučan Nenadić, Mirna Begović, Mislav Radić

**Affiliations:** 1 Nephrology and Haemodialysis Division, Internal Medicine Department, University Hospital of Split, 21000 Split, Croatia; 2 Department of Internal Medicine, School of Medicine, University of Split, 21000 Split, Croatia; 3 Nutrition and Dietetics Department, University Hospital of Split, 21000 Split, Croatia; 4 School of Medicine, University of Split, 21000 Split, Croatia; 5 Rheumatology, Allergology, and Clinical Immunology Division, Internal Medicine Department, University Hospital of Split, 21000 Split, Croatia

**Keywords:** dialysis, depression, nutritional status, body composition

## Abstract

The aim of this study was to investigate the determinants of depression and nutritional status and their associations in patients treated with hemodialysis (HD) and peritoneal dialysis (PD) and the differences according to the type of treatment. There were fifty-three patients treated with maintenance HD and twenty patients treated with PD enrolled in this cross-sectional study. Body composition, anthropometric parameters, and clinical and laboratory parameters were collected for each participant. Depression was assessed with the Beck Depression Inventory II (BDI-II) and risk of malnutrition with the Malnutrition Inflammation Score (MIS). The results suggest a positive association between the level of depression and the percentage of fat mass and obesity parameters in PD and HD participants. Muscle mass and fat-free mass in HD, phase angle (PhA), and serum albumin levels in PD were negatively associated with the value of BDI-II. Participants treated with HD were found to have higher levels of pessimism and loss of interest in sex compared with PD. There is a need to integrate multidisciplinary psychological and nutritional assessment into the usual care of dialysis patients.

## 1. Introduction

A risk of developing numerous health complications, including cardiovascular (CV) events, hypertension, electrolyte and metabolic disturbances and anemia is characteristic of patients with end-stage renal disease (ESRD) [1]. For most ESRD patients, hemodialysis (HD) or peritoneal dialysis (PD) treatment is required. This patient group also faces many dietary challenges, such as food volume restriction and micronutrient restriction [2]. Dialysis patients are at high risk of malnutrition, with a prevalence of up to 27.3% on HD and up to 20.4% on PD [3]. Sarcopenia has been associated with worse outcomes in terms of higher mortality and lower quality of life in patients treated with HD [4]. Patients on long-term dialysis are also at higher risk for developing cerebrovascular disease [5]. Some of the mechanisms underlying cerebrovascular dysfunction in dialysis patients include acute reduction in cerebral blood flow noticed in HD and oxidative stress caused by uremic toxins, vascular dysfunction, and coagulation disorders [5,6]. Depression is common in ESRD patients, both PD and HD, with a prevalence of depressive symptoms of 26.5% and is associated with increased mortality [7]. Dialysis-related depression is associated with both biological and psychosocial mechanisms; biological ones include increased levels of cytokines and neurotransmitters affected by uremia, whereas psychosocial ones involve loss of freedom, sense of well-being, dietary challenges, changes in social and professional relationships, and more [8]. Dialysis treatments, HD in particular, present a great psychosocial burden considering the extensive lifestyle and dietary restrictions required for ESRD management impact a patient’s sense of control and can result in social withdrawal [9,10]. Furthermore, depression is associated with appetite loss and reduced adherence to treatment, dietary, and fluid recommendations, which links depression to increased rates of malnutrition, morbidity, and mortality [11,12,13]. Despite all this, depression remains understudied in this specific patient population with inconsistent findings across the body of literature regarding the relationship between depression and dietary adherence in ESRD patients [10].

Thus, the aim of our study was to determine the relationship between depression and nutritional status in patients treated with HD and PD and the differences according to the type of dialysis treatment.

## 2. Materials and Methods

### 2.1. Study Design and Population

The present study was conducted according to the protocol described in our previously published study, where the methods are described in detail [14]. The only difference was in the number of participants due to the 2 participants in the HD group who did not fill out the whole depression questionnaire and were excluded for the purposes of analyzing data for this paper.

This cross-sectional study conducted with fifty-three (53) HD patients and twenty (20) PD patients was carried out at the Hemodialysis department and Outpatient clinic of the Department of Nephrology and Dialysis between February and April 2022. All included patients were stable and on dialysis treatment for longer than 3 months. HD patients on maintenance HD three times a week for 4-h were recruited before mid-week HD treatment whereas PD patients were recruited during a regular visit to the nephrologist.

Participants were excluded if they met one or more criteria: immobility; changes in dialysis modality; implanted stent, pacemaker or cardioverter-defibrillator; extremity amputation; oedema; acute infection; active malignant disease; use of corticosteroids; having COVID-19 disease or being vaccinated in a period of two months; cognitive impairment; missing BDI-II questionnaire or refusing to participate. For each participant, written, as well as verbal, consent to participate in the study was obtained. Furthermore, the protocol of the study was approved by the Ethics Committee of the University Hospital of Split, Croatia.

### 2.2. Body Composition and Anthropometric Measurement

An MC-780 Multi Frequency Segmental Body Analyzer (Tanita, Tokyo, Japan) was used to assess the body composition of the participants of our study using bioelectrical impedance analysis (BIA). The device determines the electrical resistance of different tissues using eight electrodes and a constant high-frequency current flow. It obtains data about body mass (kg), total body water (TBW; kg), extracellular water (ECW; kg), intracellular water (ICW; kg), muscle mass percentage (%), fat-free mass (kg), fat mass (kg and %), visceral fat, skeletal muscle index (SMI), trunk fat mass (kg and %), and phase angle (PhA, °). Data about the segmental distribution of fat and muscle mass were also reported. Participants were advised not to take any food or liquid at least three hours before the measurement, not to consume alcohol, eat or exercise excessively at least one day before the body composition measurement [15]. Height was measured using a stadiometer. Waist circumference (WC), hip circumference (HC), and mid-upper arm circumference (MUAC) were measured using a flexible, non-stretchable measuring tape. Body mass index (BMI), waist-to-hip ratio (WHR), and waist-to-height ratio (WHtR) were calculated.

### 2.3. Clinical and Laboratory Data

By thorough examination of the patient’s medical records, data about the duration of dialysis treatment, arterial hypertension, diabetes mellitus, and CV events were obtained. The data from the usual peripheral blood sampling, which underwent on the same day as body composition measurement, included: C-reactive protein (CRP; mg/L), serum albumin (g/L), serum hemoglobin (Hb; g/L), mean corpuscular volume (MCV, fL), total cholesterol (mmol/L), low-density lipoprotein cholesterol (LDL; mmol/L), high-density lipoprotein cholesterol (HDL; mmol/L), triglycerides (mmol/L), fasting blood glucose (FBG, mmol/L), uric acid (μmol/L), potassium (mmol/L), phosphates (mmol/L), sodium (mmol/L), calcium (mmol/L), magnesium (mmol/L), chloride (mmol/L), total iron-binding capacity (TIBC; μmol/L), creatine kinase (CK; mmol/L), and intact parathyroid hormone (iPTH; pmol/L). An immunoassay analyzer (Cobas e601, Roche Diagnostics, Penzberg, Germany) was used to measure iPTH. A sociodemographic questionnaire was used to obtain data about the level of education, employment, habitation place, income level, and physical activity level.

### 2.4. Depression Assessment

To assess the severity of depressive symptoms we used the Beck Depression Inventory-II (BDI-II) self-administered questionnaire which consists of 21 questions. The maximum score is 63, and cut-offs are depicted as: 0–13 suggesting minimal depression, 14–19 suggesting mild depression, 20–28 suggesting moderate depression, and 29–63 suggesting severe depression. Due to the low number of PDs and HDs in the groups of moderate and severe depression, a binary variable was created and a cut-off of ≥14 depicted the presence of depressive symptoms.

### 2.5. Malnutrition Assessment

Malnutrition Inflammation Score (MIS) was used to assess the severity of malnutrition. The score ranges from 0 to 30 and a higher score indicates a more severe level of malnutrition which was in more detail described in our previous study [14].

### 2.6. Charlson Comorbidity Index (CCI)

In order to predict 10-year survival in patients with multiple comorbidities, CCI was used. This method consists of 16 variables all related to the presence of different diseases and, considering the severity of the disease, each variable is awarded from one to three points. The used formula for the estimation of 10-year survival is 0.983^(eCCI × 0.9)^ with a higher score suggesting higher disease severity and mortality rate. The use of the CCI score was detailed in our previous study conducted with the same population of patients.

### 2.7. Statistical Analyses

Categorical data are represented by absolute and relative frequencies and their differences were tested by Chi-square Test. The normality of the distribution of numerical variables, which were further described by the median and the limits of the interquartile range, was tested by the Shapiro–Wilk test. Differences between the two independent groups were tested by Mann –Whitney’s U test. The correlation between numeric variables was evaluated by Spearman’s correlation coefficient ρ (rho). To find significant predictors of BDI-II, linear multivariate regression analyses were performed. All P values were two-sided with the level of significance set at an Alpha of 0.05. MedCalc^®^ Statistical Software version 20.111. was used to perform the statistical analyses. In this sample of participants (n_1_ = 53 and n_2_ = 20) for depicting the effect of 0.745 in the numerical variable difference between the groups with a significance level of 0.05, the power was 0.80 [16].

## 3. Results

We included 53 HD and 20 PD patients, out of which 64% were male, with a mean age of 64 years. The mean duration of dialysis treatment for both PD and HD was 36 months, whereas it was 46 months for HD patients and 24 months for PD patients. The median BDI-II score for all study participants was 7 (IQR 5–11). Depressive symptoms (BDI- II score ≥ 14) were present in 10 (19%) patients on HD and 2 (10%) patients on PD. Data about the general, anthropometric, and body composition parameters of our study population are presented in Table 1. Laboratory parameters and differences regarding dialysis modality are shown in Table S1. Basic characteristics and differences regarding dialysis modality were discussed in our previous study and since they are not the focus of this research will not be discussed in detail [14]. Data about sociodemographic parameters and differences regarding dialysis modality are shown in Table 2.

Due to the low number of participants having BDI-II scores in the range of moderate and severe depression, we depicted having depressive symptoms as scoring on BDI-II 14 or above. We investigated possible differences in measured parameters between participants with and without depressive symptoms. Significant differences are shown in Table 3.

Furthermore, differences in the presence of different depressive symptoms between HD and PD groups were found for pessimism (*p =* 0.002) and loss of sex interest (*p =* 0.04) which were more frequently present in the HD group (Table 4). Detailed analyses of the presence of specific depressive symptoms in both groups are presented in Table S2.

**Figure 1:**
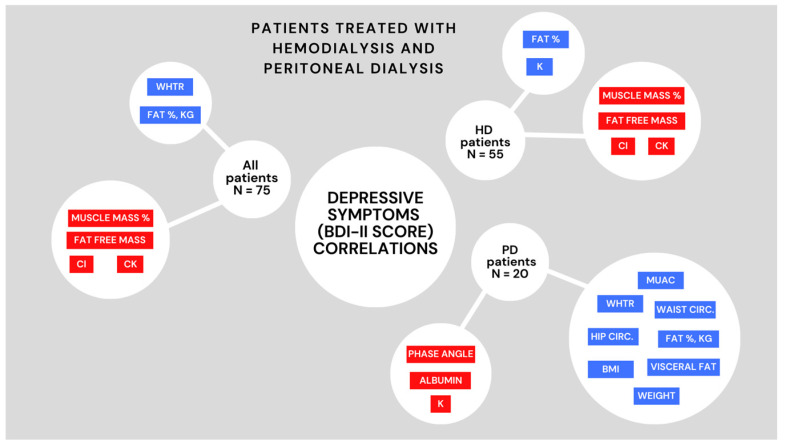
Significant correlations between depressive symptoms and measured parameters. Abbreviations: BDI-II—Beck depression inventory, HD—hemodialysis, PD—peritoneal dialysis, N—number, WHTR—waist to height ratio, Waist Circ.—waist circumference, Hip Circ.—hip circumference, MUAC—middle upper arm circumference, K—potassium, BMI- Body mass index, CK- creatine kinase, Cl—serum chloride level. Blue colour depicts positive, while red colour depicts negative correlation.

Correlations between the BDI-II score and measured parameters in HD, PD, and the total study population are shown in Table 5 and Figure 1 (only statistically significant parameters are shown).

For the total study population, positive correlations between BDI-II and muscle mass (%), fat-free mass, serum chloride and potassium level were found, and negative correlations between BDI-II and WHtR and fat mass (both % and kg) were found.

When it comes to the HD population, positive correlations between BDI-II and muscle mass (%), fat-free mass, serum chloride and potassium level were found as well, while negative correlations between BDI-II and fat percentage and serum potassium level were found. In the PD population, BDI-II correlated positively with MUAC, WHtR, WC, HC, BMI, weight, visceral fat level, and fat mass (both % and kg). On the other hand, BDI-II in the PD population negatively correlated to the levels of PhA, serum albumin, and potassium.

Results of the regression analysis are shown in Table 6. In a multivariate regression analysis with the aim to find significant predictors of BDI-II score, we found MIS score and right leg fat tissue to be positive predictors and CK to be a negative predictor of BDI-II score in all study participants. Regression analysis were also performed on the HD group to find specific predictors of BDI-II in that population. The same parameters as for the whole study population were found as predictors. Unfortunately, regression analysis were not possible for the PD population due to the low number of PD participants with depressive symptoms.

## 4. Discussion

The main results of our study show a positive association between the level of depression and the percentage of fat mass and obesity parameters in PD and HD participants. Muscle mass and fat-free mass in HD, PhA, and serum albumin level in PD were negatively associated with the value of BDI-II. 

A previous study by Heng-Jung Hsu et al. that included 209 HD patients found lower albumin levels in a group of depressed HD patients [17]. On the contrary, our results did not show a significant correlation between serum albumin levels and BDI-II score in HD participants, but a significant correlation was observed in PD participants. They also reported lower BMI levels in depressed HD participants, whereas we found no significant association between depression level and BMI in HD participants, but our results did show a positive association between BMI and BDI-II in PD participants. The possible reason for different results from our study could be a different questionnaire to assess depression used in the mentioned study and a greater number of participants. In a study by Markaki et al., a negative association was also found between depression and the level of PhA in HD patients, similarly to our PD group [8]. In the aforementioned study, no data were collected on muscle mass, fat mass, or segmental analysis of body composition. In a study of 39 patients by Ribeiro et al. [18] treated with HD, a positive correlation was found between depressive symptoms and body fat, which is similar to our results. In a cohort study by Barros et al. [19], no significant association was found between depression severity and muscle or fat mass, but they found that lean body mass had a protective effect against all-cause mortality in HD patients. 

Our regression analysis adjusted for age and sex found that MIS and percent of adipose tissue in the right leg were positive predictors of depression, and serum CK was a negative predictor of depression in HD patients. A study by Gencer et al. on 55 HD participants found a statistically significant positive correlation of BDI with MIS similar to our results [20].

A study of 55 HD patients using the Mini Nutritional Assessment short questionnaire (MNA-SF) to assess nutritional risk and the Hospital-Anxiety Depression Scale to assess depression levels showed a statistically significant increase in the frequency of depression and anxiety in patients with low MNA-SF scores [21], which is consistent with our findings regarding MIS score. All these results indicate the importance of nutritional and psychological management of ESRD patients. Higher depressive symptoms promote higher fat mass, lower muscle mass, and malnutrition.

These results cannot be interpreted causally because of the cross-sectional design, so we cannot say with certainty whether depression has a negative effect on nutritional status or whether it is the other way around.

For all study populations with lower physical activity, female gender, along with more frequent nausea, chewing or swallowing problems, and more frequent gastrointestinal symptoms in participants with BDI-II scores greater than or equal to 14 found in this study are possible reasons for inadequate nutrient intake, which, in combination with low physical activity, increases the already high risk for sarcopenia and malnutrition in this patient population. There is further evidence in the literature of associations between depression levels and dietary nonadherence in dialysis patients [10].

Differences in the frequency of various depressive symptoms in relation to the modality of dialysis show higher levels of pessimism and loss of interest in sex in participants treated with HD compared with PD. A systematic review by Budhram et al. [22] reported that PD is more preferred than HD in terms of sexual function as a quality-of-life parameter, similar to our findings. A study conducted in Africa by Tannor et al. [23] examined the differences between the quality-of-life parameters of HD and PD patients and found lower scores for symptoms, energy/fatigue, sleep, and social function, but better scores for work status and support from dialysis staff in the PD group. They noted that both dialysis patients reported sexual dysfunction. Regarding sexual dysfunction in the aforementioned study, PD patients were negatively affected by the presence of a Tenckhoff catheter protruding from the abdomen in terms of body image, while HD patients were mainly disturbed by intradialytic hypotension and orthostatic hypotension after dialysis. The higher degree of pessimism in HD patients in our study could be due to more frequent hospital visits or their older age than in PD patients. Further research is needed to better understand the differences in depressive symptoms between dialysis modalities.

The limitations of this study primarily arose from the cross-sectional design which by itself prevented any causal conclusions. Furthermore, the relatively small number of participants included, together with the age difference between the groups and different dialysis duration and the presence of comorbidities in the HD and PD groups, should be taken into consideration for possible impact on nutritional status.

## 5. Conclusions

We found positive association between the level of depression and the percentage of fat mass and obesity parameters in PD and HD participants, as well as differences between depressive symptoms between PD and HD.

Both depression and nutritional problems are influenced by many complex factors in the vulnerable group of dialysis patients and are likely to interact with each other. A possible solution to overcoming these two problems may lie in a multidisciplinary approach by integrating both psychological and nutritional assessment into the usual care of dialysis patients.

## Figures and Tables

**Table 1:** Data about general, anthropometric, and body composition parameters of our study population.

	HD (*n* = 53)	PD (*n* = 20)	*p* *
Basic characteristics			
Sex (female), N (%)	36 (68)	11 (55)	0.30
Age (years), median (IQR)	69 (56–76)	54 (36–66)	**0.002**
Dialysis duration (months), median (IQR)	46 (21–78)	24 (8.5–36)	**0.001**
Presence of AH, N (%)	34 (77)	17 (85)	0.74
Presence of DM, N (%)	14 (30)	1 (5)	**0.03**
Presence of MD, N (%)	14 (32)	0	**0.003**
Charlson Comorbidity Index, median (IQR)	6 (4–8)	4 (2–5.5)	**<0.001**
Estimated 10-year survival (%), median (IQR)	2 (0–53)	53 (15–87)	**0.001**
Anthropometric parameters			
Weight (kg), median (IQR)	73.5 (66.3–84)	80.75 (70.53–101.7)	0.06
Height (cm), median (IQR)	175 (164–183)	173 (171–178.75)	0.89
BMI (kg/m^2^), median (IQR)	24.6 (22.4–27.45)	25.65 (23.53–31.38)	0.11
Middle upper arm circumference (cm), median (IQR)	28 (26.5–31.5)	30.3 (26.63–33.75)	0.26
Waist circumference (cm), median (IQR)	94 (88.5–101.5)	98.5 (90.25–103.75)	0.22
Hip circumference (cm), median (IQR)	102 (98.5–108.5)	108.5 (97.5–114.5)	0.06
WHR, median (IQR)	0.92 (0.86–0.97)	0.9 (0.85–0.93)	0.36
WHtR, median (IQR)	0.54 (0.5–0.59)	0.56 (0.52–0.61)	0.38
Handgrip strength (kg), median (IQR)	25.7 (17.5–32.3)	31.3 (25.4–41.58)	**0.01**
Body composition			
Total body water (kg), median (IQR)	43.2 (36.55–50.75)	42.2 (38.13–48.13)	0.98
Extracellular water (kg), median (IQR)	18.2 (16.4–20.75)	19.6 (17.28–20.88)	0.36
Intracellular water (kg), median (IQR)	25 (20.4–29.75)	23.7 (20.25–28.1)	0.62
Fat mass (kg), median (IQR)	14.4 (7.25–21.05)	19.3 (12.1–29.98)	**0.01**
Fat mass (%), median (IQR)	18.5 (11.6–25.65)	24.5 (16.68–33.2)	**0.03**
Fat-free mass (kg), median (IQR)	61.9 (52.6–70.7)	62.2 (55.3–69.38)	0.65
Visceral fat level, median (IQR)	9 (8–11)	8 (4.5–13.5)	0.35
Muscle mass (%), median (IQR)	58.8 (49.95–67.2)	59.1 (52.48–65.98)	0.65
Phase angle (°), median (IQR)	4.8 (4.3–5.75)	5.2 (4.73–5.88)	**0.04**
Trunk fat mass (kg), median (IQR)	7.1 (3.4–11.3)	9.6 (6.65–17.75)	**0.02**
Trunk fat mass (%), median (IQR)	16.9 (9.5–23.9)	22.3 (16.15–31.35)	**0.01**
SMI, median (IQR)	7.93 (7.11–9.1)	8.6 (7.6–9.37)	0.25

* *p*-values were obtained with the Chi-squared Test for categorical data and the Mann–Whitney U test for non-parametric numerical data (*p* < 0.05). Abbreviations: *n*-number, IQR—interquartile range, AH—arterial hypertension, DM—diabetes mellitus, MD—malignant disease, BMI—Body Mass Index, WHR—waist-to-hip ratio, WHtR—waist-to-height ratio, SMI—sarcopenic muscle index. Bold denotes statistical significance.

**Table 2:** Sociodemographic data of study population.

	HD (*n* = 55)	PD (*n* = 20)	TOTAL (*n*= 75)	*p* *
BDI-II score, median (IQR)	7 (5–12)	6 (4–9)	7 (5–11)	0.32
Sociodemographic data, number (%)				
Education level				
Elementary school	11 (20)	4 (20)	15 (20)	0.19
Middle school	34 (62)	10 (50)	44 (59)	
Intermediate education	4 (7)	5 (25)	9 (12)	
Faculty education or higher	6 (11)	1 (5)	7 (9)	
Working status				
Employed full-time	2 (4)	8 (40)	53 (71)	**<0.001**
Unemployed	4 (7)	2 (10)	19 (25)	
Student	0	1 (5)	3 (4)	
Retired	45 (82)	9 (45)	54 (72)	
Housewife	1 (1.8)	0	37 (49)	
Sick leave	3 (5.5)	0	37 (49)	
Inhabitant of				
Village	11 (20)	7 (35)	18 (24)	0.18
Town/city	44 (80)	13 (65)	57 (76)	
Monthly household budget				
<2850 kn	8 (15)	1 (5)	9 (12)	0.42
2850–5700 kn	15 (27)	3 (16)	18 (24)	
5700–8600 kn	12 (22)	7 (37)	19 (26)	
8600–11,400 kn	11 (20)	3 (16)	14 (19)	
>11,400 kn	9 (16.4)	5 (26.3)	14 (18.9)	
Number of people in household				
1	10 (18)	2 (10)	12 (16)	0.11
2	21 (38)	8 (40)	29 (39)	
3	14 (25)	1 (5)	15 (20)	
4	4 (7)	4 (20)	8 (11)	
5	3 (5.5)	3 (15)	6 (8)	
6	2 (3.6)	2 (10)	4 (5.3)	
9	1 (2)	0	1 (1)	
Physical activity intensity				
Extremely weak	1 (2)	3 (15)	4 (5)	0.10
Weak	22 (40)	9 (45)	31 (41)	
Moderate	29 (53)	8 (40)	37 (49)	
Intense	3 (5)	0	3 (4)	

* *p*-values were obtained with the chi-square test for categorical data, and the Mann-Whitney U test for non-parametric numerical data. Abbreviations: IQR—interquartile range, BDI—II Beck Depression Index, HD—hemodialysis, PD—peritoneal dialysis, kn-kuna (Croatian currency), *n*—number. Bold denotes statistical significance.

**Table 3:** Significant differences between measured parameters regarding the presence of depressive symptoms.

	Number (%)			
	BDI-II < 14 (*n*= 61)	BDI-II ≥ 14 (*n* = 12)	Total (*n* = 73)	*p* *
Sex				
Male	43 (70)	4 (33)	47 (64)	**0.02**
Female	18 (30)	8 (67)	26 (36)	
Physical activity intensity				
Extremely weak	2 (3)	2 (17)	4 (5)	**0.03**
Weak	23 (38)	8 (67)	31 (42)	
Moderate	33 (54)	2 (17)	35 (48)	
Intense	3 (5)	0 (0)	3 (4)	
Nausea	10 (16)	6 (50)	16 (22)	**0.02**
Have chewing/swallowing problem	4 (7)	4 (33)	8 (11)	**0.02**
Gastrointestinal symptoms				
No symptoms with good appetite	48 (79)	5 (42)	53 (73)	**0.02**
Mild symptoms	11 (18)	6 (50)	17 (23)	
Frequent diarrhea or vomiting or severe anorexia	(3)	1 (8)	3 (4)	
Nutritionally related functional impairment				
Normal-to-improved functional capacity	36 (59)	0	36 (49)	**<0.001**
Occasional difficulty with baseline ambulation	24 (39)	12 (100)	36 (49)	
Bed/chair- ridden, or little to no physical activity	1 (2)	0	1 (1)	
Fat mass (kg), (IQR)	14.4 (8.6–22.2)	17.6 (13.4–40.5)	14.7 (9.3–24.7)	**0.04**
Fat mass (%), (IQR)	17.9 (12.5–27)	23.6 (20.4–37.3)	20.2 (13.7–27.8)	**0.01**
Na (umol/L)	138 (136–140)	135.5 (133.5–137)	138 (135–139)	**0.01**
Cl (umol/L)	99 (97–101)	96.5 (95–99)	99 (96–101)	**0.01**

* *p*-values were obtained with Fisher’s exact test for categorical variables and the Mann-Whitney U test for non-parametric numerical data. Abbreviations: BDI-II—Beck depression inventory, *n*-number, IQR—interquartile range, HD—hemodialysis, PD—peritoneal dialysis, Na—serum sodium level, Cl—serum chloride level. Bold denotes statistical significance.

**Table 4:** Differences between depressive symptoms in HD and PD participants.

Beck Depression Inventory (BDI-II)			
	Median (IQR)		
	HD (*n* = 53)	PD (*n* = 20)	*p* *
1. SADNESS	0 (0–0)	0 (0–0)	0.06
2. PESIMISM	1 (0–1)	0 (0–0)	**0.002**
3. PAST FAILURE	0 (0–0)	0 (0–0)	0.18
4. LOSS OF PLEASURE	0 (0–1)	0 (0–1)	0.89
5. GUILTY FEELINGS	0 (0–0)	0 (0–0)	0.64
6. PUNISHMENT FEELING	0 (0–0)	0 (0–0)	0.41
7. SELF-DISLIKE	0 (0–0)	0 (0–0)	0.14
8. SELF-CRITICALNESS	0 (0–0.5)	0 (0–1)	0.62
9. SUICIDAL THOUGHTS	0 (0–0)	0 (0–0)	0.56
10. CRYING	0 (0–0)	0 (0–0)	0.94
11. AGITATION	0 (0–1)	0 (0–1)	0.30
12. LOSS OF INTEREST	0 (0–1)	0 (0–1)	0.98
13. INDECIDIVENESS	0 (0–0.5)	0 (0–0)	0.46
14. WORTHLESSNESS	0 (0–0)	0 (0–0)	0.26
15. LOSS OF ENERGY	1 (1–1)	1 (0–1)	0.46
16. CHANGES IN SLEEP	1 (0–1)	1 (0–1)	0.42
17. IRRITABILITY	0 (0–1)	0 (0–1)	0.72
18. CHANGES IN APPETITE	0 (0–1)	1 (0–1)	0.09
19. CONCENTRATION	0 (0–1)	0 (0–1)	0.41
20. TIREDNESS	1 (1–1)	1 (0–1)	0.25
21. LOSS OF INTEREST IN SEX	1.5 (0–3)	1 (0–1)	**0.04**

* *p*-values were obtained with Mann Whitney U-test. Abbreviations: BDI-II—Beck depression inventory, *n*-number, IQR—interquartile range, HD—hemodialysis, PD—peritoneal dialysis. Bold denotes statistical significance.

**Table 5:** Significant correlations between depressive symptoms and measured parameters.

	HD (*n* = 55)	PD (*n* = 20)
Parameters	Rho (*p*-Value *)	Rho (*p*-Value *)
Middle upper arm circumference (cm)	−0.119 (0.40)	**0.506 (0.03)**
Waist circumference (cm)	−0.096 (0.50)	**0.495 (0.04)**
Hip circumference (cm)	0.127 (0.38)	**0.501 (0.03)**
WHtR	0.174 (0.22)	**0.503 (0.03)**
Height (cm)	**−0.430 (<0.001)**	−0.242 (0.33)
Weight (kg)	−0.210 (0.13)	**0.533 (0.02)**
BMI (kg/m^2^)	0.080 (0.57)	**0.508 (0.03)**
Fat mass (%)	**0.319 (0.02)**	**0.547 (0.02)**
Fat mass (kg)	0.219 (0.12)	**0.528 (0.02)**
Visceral fat	0.042 (0.77)	**0.479 (0.04)**
Fat-free mass (kg)	**−0.398 (<0.001)**	0.184 (0.47)
Muscle mass (%)	**−0.398 (<0.001)**	0.184 (0.47)
Phase angle (°)	**−0.32 (0.02)**	**−0.489 (0.04)**
Creatine kinase (mmol/L)	**−0.476 (<0.001)**	0 (>0.99)
Serum albumin (g/L)	0.064 (0.65)	**−0.527 (0.02)**
Potassium (mmol/L)	**0.284 (0.04)**	**−0.506 (0.03)**
Chloride (mmol/L)	**−0.340 (0.02)**	−0.376 (0.14)

* *p*-values were obtained with Spearman’s coefficient of rank correlation Rho. Abbreviations: HD—hemodialysis, PD—peritoneal dialysis, *n*—number, WHtR—waist to height ratio, BMI—body mass index. Bold denotes statistical significance.

**Table 6:** Predictors of BDI-II score in HD patients.

Predictors	BDI-II Multivariate Linear Regression			
	Adjusted for Age and Sex			
	ß	*p* *	95% CI	
HD				
RL FatP	0.23	**0.003**	0.081–0.376	R = 0.612 R^2^ = 0.374 Corrected R^2^ = 0.329
MIS score	0.96	**0.004**	0.322–1.605	
CK	−0.04	**0.03**	−0.071–(−0.004)	
**PD**	-	-	-	

* *p*-values were obtained with multivariate stepwise linear regression analysis. Abbreviations: BDI-II—Beck Depression Inventory-II, HD—hemodialysis, PD—peritoneal dialysis, RL FatP—right leg fat percentage, MIS—Malnutrition Inflammation Score, CK—creatine kinase, CI—confidence interval. Bold denotes statistical significance.

## Data Availability

Data is available at corresponding author e-mail upon request.

## Supplementary Materials

The following supporting information can be downloaded at: https://www.mdpi.com/article/10.3390/ijerph20032285/s1, Table S1: Laboratory parameters and differences regarding dialysis modality, Table S2: Detailed analyses of the presence of specific depressive symptoms.

## References

[1] Jankowski J., Floege J., Fliser D., Böhm M., Marx N. (2021). Cardiovascular Disease in Chronic Kidney Disease. Circulation.

[2] Kalantar-Zadeh K., Tortorici A.R., Chen J.L.T., Kamgar M., Lau W.-L., Moradi H., Rhee C.M., Streja E., Kovesdy C.P. (2015). Dietary Restrictions in Dialysis Patients: Is There Anything Left to Eat?. Semin. Dial..

[3] Naini A.E., Karbalaie A., Abedini M., Askari G., Moeinzadeh F. (2016). Comparison of Malnutrition in Hemodialysis and Peritoneal Dialysis Patients and Its Relationship with Echocardiographic Findings. J. Res. Med. Sci..

[4] Kim J.-K., Kim S.G., Oh J.-E., Lee Y.-K., Noh J.-W., Kim H.J., Song Y.R. (2019). Impact of Sarcopenia on Long-Term Mortality and Cardiovascular Events in Patients Undergoing Hemodialysis. Korean J. Intern. Med..

[5] Liabeuf S., Pepin M., Franssen C.F.M., Viggiano D., Carriazo S., Gansevoort R.T., Gesualdo L., Hafez G., Malyszko J., Mayer C. (2021). Chronic Kidney Disease and Neurological Disorders: Are Uraemic Toxins the Missing Piece of the Puzzle?. Nephrol. Dial. Transplant..

[6] Masson P., Webster A.C., Hong M., Turner R., Lindley R.I., Craig J.C. (2015). Chronic Kidney Disease and the Risk of Stroke: A Systematic Review and Meta-Analysis. Nephrol. Dial. Transplant..

[7] Palmer S., Vecchio M., Craig J.C., Tonelli M., Johnson D.W., Nicolucci A., Pellegrini F., Saglimbene V., Logroscino G., Fishbane S. (2013). Prevalence of Depression in Chronic Kidney Disease: Systematic Review and Meta-Analysis of Observational Studies. Kidney Int..

[8] Markaki A.G., Charonitaki A., Psylinakis E., Dimitropoulakis P., Spyridaki A. (2019). Nutritional Status in Hemodialysis Patients Is Inversely Related to Depression and Introversion. Psychol. Health Med..

[9] Finnegan-John J., Thomas V.J. (2013). The Psychosocial Experience of Patients with End-Stage Renal Disease and Its Impact on Quality of Life: Findings from a Needs Assessment to Shape a Service. ISRN Nephrol..

[10] Gebrie M.H., Ford J. (2019). Depressive Symptoms and Dietary Non-Adherence among End Stage Renal Disease Patients Undergoing Hemodialysis Therapy: Systematic Review. BMC Nephrol..

[11] Cukor D., Friedman S. (2004). Number 2 Towards the Psychosocial Treatment of Depressed Patients on Dialysis. Internet J. Nephrol..

[12] Lee E. (2022). The Importance of Psychiatric Disorders in End-Stage Kidney Disease Patients. Kidney Res. Clin. Pract..

[13] Suseł J., Batycka-Baran A., Reich A., Szepietowski J. (2014). Uraemic Pruritus Markedly Affects the Quality of Life and Depressive Symptoms in Haemodialysis Patients with End-Stage Renal Disease. Acta Derm. Venereol..

[14] Kolak E., Radić J., Vučković M., Nenadić D.B., Begović M., Radić M. (2022). Nutritional and Hydration Status and Adherence to Dietary Recommendations in Dalmatian Dialysis Patients. Nutrients.

[15] Oki Data Corporation (2015). Mc780—User Manual.

[16] Kohn M.A., Senyak J. (2021). Sample Size Calculators. UCSF CTSI. https://sample-size.net/.

[17] Hsu H.-J., Yen C.-H., Chen C.-K., Wu I.-W., Lee C.-C., Sun C.-Y., Chang S.-J., Chou C.-C., Hsieh M.-F., Chen C.-Y. (2013). Association between Uremic Toxins and Depression in Patients with Chronic Kidney Disease Undergoing Maintenance Hemodialysis. Gen. Hosp. Psychiatry.

[18] Ribeiro H., Ferreira T., Duarte M., Baião V., Inda-Filho A., Ferreira A. (2021). Associação Entre Os Sintomas Depressivos e a Gordura Corporal Em Pacientes Com Doença Renal Crônica Em Hemodiálise. Rev. Psicol. Divers. Saúde.

[19] Barros A., da Costa B.E., Mottin C.C., d’Avila D.O. (2016). Depression, Quality of Life, and Body Composition in Patients with End-Stage Renal Disease: A Cohort Study. Rev. Bras. Psiquiatr..

[20] Gencer F., Yıldıran H., Erten Y. (2019). Association of Malnutrition Inflammation Score With Anthropometric Parameters, Depression, and Quality of Life in Hemodialysis Patients. J. Am. Coll. Nutr..

[21] Cindoğlu Ç., Beyazgül B. (2021). Nutritional Status and Anxiety-Depression Relationship in Hemodialysis Patients. J. Surg. Med..

[22] Budhram B., Sinclair A., Komenda P., Severn M., Sood M.M. (2020). A Comparison of Patient-Reported Outcome Measures of Quality of Life By Dialysis Modality in the Treatment of Kidney Failure: A Systematic Review. Can. J. Kidney Health Dis..

[23] Tannor E.K., Archer E., Kapembwa K., van Schalkwyk S.C., Davids M.R. (2017). Quality of Life in Patients on Chronic Dialysis in South Africa: A Comparative Mixed Methods Study. BMC Nephrol..

