# Supplementary material for: Body Composition Parameters Correlate to Depression Symptom Levels in Patients Treated with Hemodialysis and Peritoneal Dialysis
Source: Int J Environ Res Public Health. 2023 Jan 27;20(3):2285. doi: 10.3390/ijerph20032285 (PMC9915081; doi:10.3390/ijerph20032285)
Supplement: Supplementary file 1 [file ijerph-20-02285-s001.zip › ijerph-2154289-supplementary.pdf]

Table S1. Laboratory parameters and differences regarding dialysis modality

	HD (n = 53) Median (IQR)	PD (n = 20) Median (IQR)	*p**
Hb (g/L)	116 (111 - 120)	115.5 (100.75 - 122.75)	0.92
MCV (fL)	93.9 (89.4 - 98.4)	88.75 (87 - 93.38)	0.02
Iron (mol/L)	12 (10 - 14)	14 (10 - 18)	0.44
TIBC (mol/L)	40 (36 - 47)	44 (39.75 - 52.5)	0.03
FBG (mmol/L)	6.2 (4.9 - 7.5)	5.55 (4.88 - 6)	0.04
Uric acid (mmol/L)	335 (295.5 - 369.5)	329.5 (279.5 - 362.5)	0.56
Total cholesterol (mmol/L)	3.7 (3 - 4.4)	4.5 (3.6 - 6.8)	0.02
Triglycerides (mmol/L)	1.7 (1.1 - 2.3)	1.8 (0.8 - 4.3)	0.65
HDL cholesterol (mmol/L)	1 (0.9 - 1.3)	1.2 (0.9 - 1.3)	0.61
LDL cholesterol (mmol/L)	1.7 (1.2 - 2.5)	2.7 (2 - 3.5)	0.03
Serum albumin (g/L)	41.8 (39.9 - 43.7)	38.1 (35.8 - 40.5)	<0.001
Sodium (mmol/L)	137 (135 - 139)	139 (134.3 - 140)	0.47
Potassium (mmol/L)	5.5 (5 - 6)	4.3 (4.1 - 4.9)	<0.001
Chloride (mmol/L)	99 (97.8 - 101)	97 (95 - 101)	0.07
Calcium (mmol/L)	2.2 (2.2 - 2.3)	2.2 (2.1 - 2.4)	0.96
Phosphate (mmol/L)	1.7 (1.3 - 2.1)	1.8 (1.5 - 1.9)	0.46
Magnesium (mmol/L)	1.04 (0.98 - 1.15)	1 (0.6 - 1.2)	0.75
CRP (mg/L)	3.4 (1.4 - 9.7)	2.6 (1.8 - 10.2)	0.82
iPTH (pmol/L)	26.8 (14.85 - 48.53)	23.2 (7 - 90.3)	0.96

*Mann Whitney U test

*p-values were obtained with the Mann–Whitney U test ($p < 0.05$). Abbreviations: N—number. IQR—interquartile range. Hb—hemoglobin. MCV—Mean Corpuscular Volume. TIBC—total iron-binding capacity. FBG—fasting blood glucose. HDL—high-density lipoproteins. LDL—low-density lipoproteins. CRP—C-reactive protein. iPTH—intact parathyroid hormone.

Table S2. Detailed analyses of the presence of specific depressive symptoms

ALL PARTICIPANTS	Number of participants (%)			
	0	1	2	3
1.SADNESS	62 (90)	6 (8,5)	3 (4,2)	0
2. PESIMISM	50 (70,4)	14 (19,7)	4 (5,6)	3 (4,2)
3. PAST FAILURE	60 (84,5)	10 (14,1)	1 (1,4)	0
4. LOSS OF PLEASURE	44 (62)	20 (28,2)	6 (8,5)	1 (1,4)
5. GUILTY FEELINGS	65 (91,5)	6 (8,5)	0	0
6. PUNISHMENT FEELING	69 (97,2)	1 (1,4)	0	1 (1,4)
7. SELF-DISLIKE	65 (91,5)	6 (8,5)	0	0
8. SELF-CRITICALNESS	53 (74,6)	16 (22,5)	2 (2,8)	0
9. SUICIDAL THOUGHTS	70 (98,6)	1 (1,4)	0	0
10. CRYING	60 (84,5)	6 (8,5)	1 (1,4)	4 (5,6)
11. AGITATION	47 (66,2)	23 (32,4)	0	1 (1,4)
12. LOSS OF INTEREST	44 (62)	26 (36,6)	0	1 (1,4)
13. INDECIDIVENESS	55 (77,5)	14 (19,7)	0	2 (2,8)
14. WORTHLESSNESS	61 (85,9)	7 (9,9)	3 (4,2)	0
15. LOSS OF ENERGY	16 (22,5)	40 (56,3)	14 (19,7)	1 (1,4)
16. CHANGES IN SLEEP	23 (32,4)	36 (50,7)	8 (11,3)	4 (5,6)
17. IRRITABILITY	49 (70)	21 (30)	0	0
18. CHANGES IN APPETITE	45 (63,4)	23 (32,4)	1 (1,4)	2 (2,8)
19. CONCENTRATION	46 (64,8)	23 (32,4)	2 (2,8)	0
20. TIREDNESS	16 (22,5)	45 (63,4)	9 (12,7)	1 (1,4)
21. LOSS OF INTEREST IN SEX	22 (31)	25 (35,2)	9 (12,7)	15 (21,1)

PARTICIPANTS ON HD	Number of participants (%)			
	0	1	2	3
1.SADNESS	44 (80)	6 (11,3)	3 (5,7)	0
2. PESIMISM	32 (60,4)	14 (26,4)	4 (7,5)	3 (5,7)
3. PAST FAILURE	43 (81,1)	9 (17)	1 (1,9)	0
4. LOSS OF PLEASURE	32 (60,4)	17 (32,1)	4 (7,5)	0
5. GUILTY FEELINGS	49 (92,5)	4 (7,5)	0	0
6. PUNISHMENT FEELING	51 (96,2)	1 (1,9)	0	1 (1,9)
7. SELF-DISLIKE	47 (88,7)	6 (11,3)	0	0
8. SELF-CRITICALNESS	40 (75,5)	13 (24,5)	0	0
9. SUICIDAL THOUGHTS	52 (98,1)	1 (1,9)	0	0
10. CRYING	45 (84,9)	4 (7,5)	0	4 (7,5)
11. AGITATION	37 (69,8)	15 (28,3)	0	1 (1,9)
12. LOSS OF INTEREST	33 (62,3)	19 (35,8)	0	1 (1,9)
13. INDECIDIVENESS	40 (75,5)	11 (20,8)	0	2 (3,8)
14. WORTHLESSNESS	44 (83)	7 (13,2)	2 (3,8)	0
15. LOSS OF ENERGY	11 (20,8)	30 (56,6)	11 (20,8)	1 (1,9)
16. CHANGES IN SLEEP	17 (32,1)	25 (47,2)	7 (13,2)	4 (7,5)
17. IRRITABILITY	37 (71,2)	15 (28,8)	0	0
18. CHANGES IN APPETITE	37 (69,8)	13 (24,5)	1 (1,9)	2 (3,8)
19. CONCENTRATION	33 (62,3)	18 (34)	2 (3,8)	0
20. TIREDNESS	10 (18,9)	35 (66)	7 (13,2)	1 (1,9)
21. LOSS OF INTEREST IN SEX	15 (28,3)	16 (30,2)	7 (13,2)	15 (28,3)

Abbreviations: HD- hemodialysis

PARTICIPANTS ON PD	Number of participants (%)			
	0	1	2	3
1.SADNESS	18 (100)	0	0	0
2. PESIMISM	18 (100)	0	0	0
3. PAST FAILURE	17 (94,4)	1 (5,6)	0	0
4. LOSS OF PLEASURE	12 (66,7)	3 (16,7)	2 (11,1)	1 (5,6)
5. GUILTY FEELINGS	16 (88,9)	2 (11,1)	0	0
6. PUNISHMENT FEELING	18 (100)	0	0	0
7. SELF-DISLIKE	18 (100)	0	0	0
8. SELF-CRITICALNESS	13 (72,2)	3 (16,7)	2 (11,1)	0
9. SUICIDAL THOUGHTS	18 (100)	0	0	0
10. CRYING	15 (83,3)	2 (11,1)	1 (5,6)	0
11. AGITATION	10 (55,6)	8 (44,4)	0	0
12. LOSS OF INTEREST	11 (61,1)	7 (38,9)	0	0
13. INDECIDIVENESS	15 (83,3)	3 (16,7)	0	0
14. WORTHLESSNESS	17 (94,4)	0	1 (5,6)	0
15. LOSS OF ENERGY	5 (27,8)	10 (55,6)	3 (16,7)	0
16. CHANGES IN SLEEP	6 (33,3)	11 (61,1)	1 (5,6)	0
17. IRRITABILITY	12 (66,7)	6 (33,3)	0	0
18. CHANGES IN APPETITE	8 (44,4)	10 (55,6)	0	0
19. CONCENTRATION	13 (72,2)	5 (27,8)	0	0
20. TIREDNESS	6 (33,3)	10 (55,6)	2 (11,1)	0
21. LOSS OF INTEREST IN SEX	7 (38,9)	9 (50)	2 (11,1)	0

Abbreviations: PD- peritoneal dialysis
